# Detection of Rapalog-Mediated Therapeutic Response in Renal Cancer Xenografts Using ^64^Cu-bevacizumab ImmunoPET

**DOI:** 10.1371/journal.pone.0058949

**Published:** 2013-03-14

**Authors:** Albert J. Chang, Rebecca Sohn, Zhi Hong Lu, Jeffrey M. Arbeit, Suzanne E. Lapi

**Affiliations:** 1 Department of Radiation Oncology, Washington University, St. Louis, Missouri, United States of America; 2 Department of Radiology, Washington University, St. Louis, Missouri, United States of America; 3 Urology Division, Department of Surgery, Washington University, St. Louis, Missouri, United States of America; 4 Siteman Cancer Center, St. Louis, Missouri, United States of America; City of Hope, United States of America

## Abstract

The importance of neovascularization for primary and metastatic tumor growth fostered numerous clinical trials of angiogenesis inhibitors either alone or in combination with conventional antineoplastic therapies. One challenge with the use of molecularly targeted agents has been the disconnection between size reduction and tumor biologic behavior, either when the drug is efficacious or when tumor resistance emerges. Here, we report the synthesis and characterization of ^64^Cu-NOTA-bevacizumab as a PET imaging agent for imaging intratumoral VEGF content in vivo. ^64^Cu-NOTA-bevacizumab avidly accumulated in 786-O renal carcinoma xenografts with lower levels in host organs. RAD001 (everolimus) markedly attenuated ^64^Cu-NOTA-bevacizumab accumulation within 786-O renal carcinoma xenografts. Tumor tissue and cellular molecular analysis validated PET imaging, demonstrating decreases in total and secreted VEGF content and VEGFR2 activation. Notably, ^64^Cu-NOTA-bevacizumab PET imaging was concordant with the growth arrest of RAD001 tumors. These data suggest that immunoPET targeting of angiogenic factors such as VEGF could be a new class of surrogate markers complementing the RECIST criteria in patients receiving molecularly targeted therapies.

## Introduction

Angiogenesis, the growth of new blood vessels, is a hallmark of cancer promoting tumor growth, invasion, and metastasis [1]. Nascent tumors are supported by oxygen and nutrients from nearby blood vessels, however, as the tumor grows, the blood supply becomes insufficient and several signaling pathways stimulate neovascularization expansion [2]. Neovessels may also act as tumor metastatic conduits [2]. The apparent importance of neovascularization for primary and metastatic tumor growth fostered numerous angiogenesis inhibitor clinical trials either alone or in combination with conventional antineoplastic therapies [3], [4]. These agents delayed tumor growth with initial improvements in therapeutic efficacy associated with vascular network normalization [4]. However, not all patients respond to anti-angiogenic therapy, and resistance almost invariably develops despite initial improvement. Preclinical studies have suggested that angiogenesis inhibitors increase tumor invasiveness and metastasis [5], though this clinical aggressiveness enhancement has yet to be clearly seen in patients. As such, a better understanding of the limitations and acquired resistance to angiogenesis inhibitors is necessary. Testing therapy-induced angiogenic factor secretion reduction offers the promise of early identification of responsive patients, and more rapid detection of agent-specific resistance emergence.

Vascular Endothelial Growth Factor (VEGF) plays a central role in angiogenesis and has emerged as a prominent therapeutic target. VEGF expression is induced in malignancies by several mechanisms. At the transcription level, VEGF is a major target of the heterodimeric hypoxia-inducible factors (HIFs) [6]. HIFs are composed of, unstable alpha (HIF-1α, HIF-2α, HIF-3α) and constitutively expressed beta (HIF-1β) subunits [6]. In normoxia, prolyl and asparaginyl hydroxylases create binding sites for the E3 ubiquitin ligase von Hippel Lindau (VHL) protein and inhibit HIF transcriptional activity, respectively. During hypoxia, the oxygen-dependent hydroxylases are inhibited, HIF1/2 transcription factors are stabilized, and angiogenic, metabolic, and stem cell target genes are induced. In addition to VEGF, HIF transcription factors upregulate multiple angiogenic factors [7]. However, recent data in a nondisease model of HIF-1 gain of function demonstrates that VEGF is the most important for neovascular induction [8]. As loss of VHL function underlies clear cell renal carcinoma development [9], these tumors are particularly hypervascular due to HIFα-mediated induction of multiple angiogenic factors including VEGF [6].

In addition to transcription factor overexpression, the phosphoinositide 3-kinase (PI3K) pathway is a parallel module regulating HIF- and VEGF-dependent tumor cell angiogenic factor production [10]. The PI3K pathway is hyperactivated in the majority of human cancers due to multiple mechanisms [11]. Mammalian target of rapamycin (mTOR) is a serine-threonine kinase downstream of PI3K. mTOR resides within two complexes localized in distinct intracellular compartments and each possessing specific functions [12], [13]. mTORC1 regulates protein synthesis at multiple levels including translational initiation and ribosome biogenesis [14]. The HIFα subunits and VEGF are mTORC1 translational targets, and are functional in normoxic malignant cells with PI3K activation [15]. mTORC2 modulates multiple cellular and secondary microenvironmental functions including cell survival, motility, proliferation, and angiogenesis via its targets AKT, SGK, and PKC, and HIF-2α. As PI3K and mTOR are also downstream of VEGFR2, the principal VEGF receptor signaling in endothelial cells [16], mTOR has a potential dual neovascularization function in both tumor and endothelial cells.

Due to its near ubiquitous upregulation, there has been intense clinical interest in mTOR pathway targeting in solid malignancies. Rapamycin and its analogs, everolimus, temserolimus, and deforlimus, (rapalogs), bind to the cyclophilin, FKBP-12, forming a complex inhibiting mTORC1 [17]. mTORC2 activity is inhibited with prolonged rapalog exposure in some cell lines [18], likely due to newly synthesized mTOR sequestration in inactive rapalog complexes. In early preclinical studies, rapamycin was shown to decrease both tumor growth and neovascularization [19]. In other preclinical studies, everolimus inhibited tumor growth and VEGF expression [17]. Due to promising Phase III efficacy data, rapalogs have been approved for treatment of patients with metastatic renal cell cancer (RCC) [20]. However, therapeutic resistance either is present at the onset or also develops during rapalog treatment [21]. Several recent and past publications have highlighted either bypass signaling, or genetic gain of function of mTOR downstream targets [22]–[24].

As VEGF is a downstream mTOR activation marker and a major driver of angiogenesis, its expression level might qualify as a rapalog sensitivity marker. Bevacizumab is a humanized monoclonal antibody that binds to all VEGF isoforms [25]. Previous studies have suggested that radiolabeled bevacizumab can noninvasively image and quantify VEGF expression [26]–[28]. Although ^64^Cu-DOTA(1,4,7,10 tetraazacyclododecane-1,4,7,10-tetraacetic acid)-bevacizumab showed promising results for preclinical VEGF xenograft imaging [28], enhanced liver accumulation associated with the known in vivo instability of copper-DOTA, motivated us to investigate a more stable copper chelate. Recent work by Zhang *et al.* using NOTA(1,4,7,10 tetraazacyclonoane-N,N’,N”,-triacetic acid) as a chelate for ^64^Cu-bevacizumab illustrated the superiority of this complex for PET imaging [29]. Here we used and evaluated the VEGF specific PET tracer ^64^Cu-NOTA-bevacizumab to monitor changes to rapalog administration. We first tested the imaging efficacy of ^64^Cu-NOTA-bevacizumab in renal carcinoma xenografts, a tumor with marked VEGF overexpression. Next, we tested ^64^Cu-NOTA-bevacizumab as an mTOR inhibition tumor response indicator. Our study demonstrates that this antibody-conjugated PET tracer may ultimately be useful both to select and continue patients on molecular therapies targeting secreted angiogenic growth factors.

## Materials and Methods

### Synthesis of ^64^Cu-NOTA-bevacizumab

^64^Cu was produced via the ^64^Ni(p,n)^64^Cu nuclear reaction using the CS-15 cyclotron (Cyclotron Corporation, Berkeley, CA) and separated via ion exchange as previously described [30]. Bevacizumab (Avastin™, Genentech/Roche, South San Francisco, CA) was incubated in a 1∶5 molar ratio with 2-(pisothiocyanatobenzyl)-1,4,7-triazacyclononane-1,4,7-triacetic acid (SCN-Bz-NOTA) (Macrocyclics, Dallas, TX) in 0.1 M NaHCO_3_ buffer pH 9.0 for 30 min. The resulting product, NOTA-bevacizumab, was purified via Zeba Spin Desalting Columns (Pierce Biotechnology, Rockford, IL). ^64^Cu was complexed with NOTA-bevacizumab at a ratio of 666 MBq/mg (18 mCi/mg) of antibody in 0.1 M NH_4_OAc buffer pH 5.5 at 37°C for 1h with constant agitation. ^64^Cu-NOTA-bevacizumab was purified using Zeba Spin Desalting Columns (Pierce Biotechnology, Rockford, IL) and radiochemical purity was determined by analytical size-exclusion chromatography (Superose 12 10/300 GL, GE Healthcare, Piscataway, NJ) with 20 mM HEPES and 150 mM NaCl (pH 7.3) eluted at a flow rate of 0.75 mL/min. Millenium 32 software (Waters, Milford, MA) was used to quantify chromatograms by integration. ^64^Cu-NOTA-bevacizumab was incubated at 37°C with human serum for 48 hours and evaluated for stability with size-exclusion chromatography.

### Radiotracer Binding Kinetics

VEGF_165_ (R&D Systems, Minneapolis, MN) was serially diluted in 0.5-fold concentrations ranging from 10 µg/mL to 0.4 µg/mL in bicarbonate coating buffer (5 mM Na_2_CO_3_, 35 mM NaHCO_3,_ pH 9.6). 50 µl of VEGF solution at each concentration was pipetted in triplicate to the wells of a 96-well plate and incubated overnight at 4°C. The plate was washed 3 times with 200 µl PBS followed by the addition of 100 µl of 1% BSA (Sigma, St. Louis, MO) in PBS to block the remaining protein-binding sites in each well. After overnight incubation at 4°C, each well was washed 4 times with 200 µl PBS. 74 kBq/0.1 µg (2 µCi) of ^64^Cu-NOTA-bevacizumab in 100 µl of PBS was added to each well and incubated at room temperature for 2 hours. Unbound ^64^Cu-NOTA-bevacizumab was carefully removed, each well was washed 3 times with 200 µl PBS +0.1% Tween 80 (Sigma, St. Louis, MO), then 200 µl of 0.2 N NaOH solution was added to each well and incubated for 15 minutes. Radioactivity was collected for each well and counted on a gamma counter.

### Tumor Xenograft Model

786-O (CRL-1932, ATCC, Manassas, VA) renal cell carcinoma cells were grown in RPMI media supplemented with 10% fetal bovine serum, and 1% penicillin/streptomycin. Log phase cells were harvested, resuspended in media at 1×10^5^/µl, and 1×10^6^ cells were injected subcutaneously into the ears of 6–8 week old athymic NCr-nu/nu mice (NCI, Frederick, MD). Tumors were imaged 3 weeks following injection at a tumor size of 94.9 mm^3^ (n = 4 mice per group). All animal experiments were conducted in accordance with the NIH Guidelines for the Care and Use of Research Animals and with approval of Washington University’s Animal Studies Committee.

### Radiotracer Biodistribution Studies

*In vivo* biodistribution studies were performed with athymic nude mice (n = 4 mice) to determine the uptake of ^64^Cu-NOTA-bevacizumab in 786-O renal cell cancer xenografts in relation to normal organs. ^64^Cu-bevacizumab, 0.555 MBq/0.83 µg,15 µCi, was injected i.v., mice sacrificed 24 hours later, and specific uptake of tumors and select organs was measured using a gamma counter with background and decay correction. Specific uptake was expressed as % injected dose per gram of tissue (%ID/g) as calculated by normalization to the total activity injected, using a known amount of injected activity as a standard.

### Small Animal PET Studies

Small animal PET/CT experiments were performed with the Inveon MicroPET/CT scanner (Siemens, Knoxville, TN). ^64^Cu-bevacizumab (2.96–3.7 MBq/4.4–5.5 µg (80–100 µCi) in 100 µL 0.9% sterile saline) was injected i.v. Twenty four hours later, mice were anesthetized with 2% isoflurane and imaged. Twenty minute static images (n = 4 mice for each treatment group) were collected and co-registered with image display software (Inveon Research Workplace, Siemens, Knoxville, TN). Regions of interest including the tumor and muscle were contoured, and the standard uptake values (SUV) for tumors were determined using the formula: SUV = [(MBq/mL)×(animal wt. (g))/injected dose (MBq)].

### RAD001 Treatment

RAD001 (Everolimus) emulsion (Novartis, San Diego, CA) was administered by daily gavage at 10 mg/kg of body weight. Control animals received a placebo emulsion (Novartis proprietary formulation). After the initial imaging experiments described above, mice were administered RAD001 or vehicle for 7 consecutive days and subsequently re-imaged with ^64^Cu-NOTA-bevacizumab (2.96–3.7 MBq/4.4–5.5 µg) as described above (RAD001, n = 4, vehicle, n = 4 mice). Biodistribution studies (as described above) were performed after completion of the small animal PET imaging on Day 7. For tumor growth experiments, a separate cohort of 8–12 week old mice bearing bilateral ear tumors were treated with vehicle (n = 7 mice, 14 tumors analyzed) or RAD001 (n = 9 mice, 18 tumors). Ear tumor growth was determined by measurement of the three greatest perpendicular dimensions and the tumor volume in mm^3^ was analyzed for each tumor on days 7 and 14 of treatment.

### Immunoblotting

RAD001 (n = 3 mice) and vehicle control (n = 3 mice) 786-O ear tumors were harvested, homogenized in RIPA buffer (50 mM Tris at pH 7.4, 150 mM NaCl, 1 mM EDTA, 1% nonylphenyl-polyethylene glycol (Nonidet P-40 substitute), 0.1% SDS, 1% sodium deoxycholate, 1% Triton X-100) supplemented with Protease Inhibitor Cocktail and Phosphatase Inhibitor Cocktails 1 and 2 (all 1∶50; Sigma-Aldrich, St. Louis, MO). Cleared tumor lysates were quantified for protein concentration using the BCA assay kit (Pierce Biotechnology, Rockford, IL) and stored at −80°C. Total protein (120 µg) was separated on SDS polyacrylamide gels, transferred to PVDF membranes (Invitrogen, Carlsbad, CA), blocked with 10% non-fat dry milk in Tris-buffered saline (pH 7.6) containing 0.5% Tween 20 (TBST), and probed with rabbit antibodies for S6K^T389^, S6K, AKT^S473^, AKT, pVEGFR2^Y1175^, VEGFR2 (all 1∶1,000, Cell Signaling Technology), chicken polyclonal VEGF antibody (1∶2,500 Ab14078, Abcam), and rabbit polyclonal for β-tubulin (1∶35,000, Abcam). After overnight incubation at 4°C, membranes were washed three times in TBST, incubated for 1 hour at room temperature in horseradish peroxidase-linked secondary antibodies (1∶5,000, Santa Cruz Biotechnology, Santa Cruz, CA), and protein bands were visualized with ECL plus reagent (GE Healthcare, Piscataway, NJ). Protein loading was normalized to β-tubulin.

### VEGF ELISA

786-O cells were seeded into 6-well plates at a cell density of 3×10^5^ cells per well and cultivated in complete medium overnight. Log-phase cells were then cultured in serum-free RPMI 1640 medium supplemented with vehicle (DMSO) or 100 nM or 1,000 nM rapamycin for 6 hours, the medium was replaced by 2 ml of fresh serum-free medium containing vehicle or the same different rapamycin concentrations, and the cell culture supernatant was harvested 18 hr later. Secreted VEGF protein in conditioned medium were quantified by ELISA in four independent cultures per group using the Human VEGF Quantikine ELISA Kit (R&D, Minneapolis, MN) and normalized to total cellular DNA content.

### Statistical Analysis

Data are presented as mean ± SD. The two-tailed Student’s *t*-test, the Mann-Whitney U test, one- and two-way ANOVA, and linear regression analysis (GraphPad Prism 6.0b, La Jolla, CA) were used for significance testing. A p value of <0.05 was considered statistically significant.

## Results

### Synthesis and in vitro Determination of ^64^Cu-NOTA-Bevacizumab VEGF Binding Affinity

As previous studies using ^64^Cu-DOTA conjugates showed high liver radioactivity accumulation indicating potential transmetallation to other proteins in vivo [28], [31], we opted to use NOTA which forms a more stable copper complex [32]. Bevacizumab was conjugated to NOTA-Bz-NCS and radiolabeled with ^64^Cu. Radiolabeling efficiency was 92.1%±4.7%, radiochemical purity was 98.1±1.7%, and specific activity was approximately 644 MBq/mg (17.4 mCi/mg) (n = 5 independent experiments). ^64^Cu-NOTA-bevacizumab was stable up to 48 hours in serum at 37°C with no visible degradation products on size-exclusion HPLC analysis.

**Figure 1:**
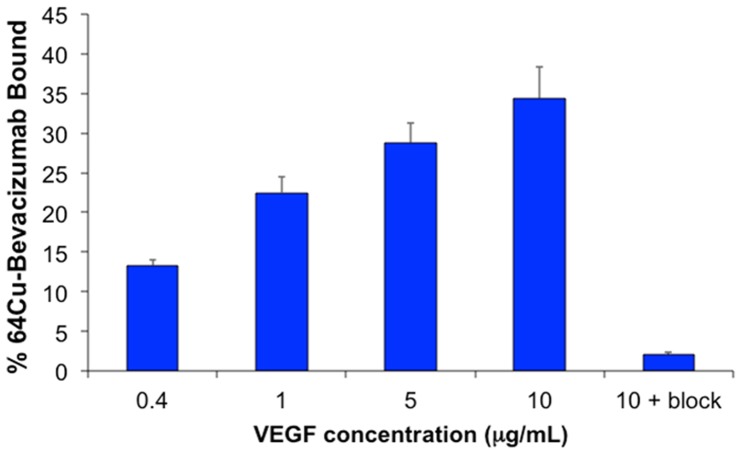
^64^Cu-NOTA-bevacizumab immunoreactivity. Increased binding of ^64^Cu-NOTA-bevacizumab was observed with increasing concentration of VEGF. The inhibitable binding blockade using 20 µg unlabeled bevacizumab highlights the tracer specificity. All assays were conducted in triplicate. Bars shown are +/− standard deviation.

To test ^64^Cu-NOTA-bevacizumab VEGF specificity immunoreactivity assays were performed. VEGF_165_ was plated in triplicate in incremental concentrations ranging from 0.4 to 10 µg/mL. The amount of bound^ 64^Cu-NOTA-bevacizumab as a percentage of total activity added was elevated with increasing concentration from 13.3±0.72% at 0.4 µg/mL VEGF_165_ to 34.4±3.89% with 10 µg/mL VEGF (Figure 1). Co-incubation with 20 µg excess unlabeled bevacizumab markedly decreased ^64^Cu-NOTA-bevacizumab VEGF_165_ binding to 2.04±0.35% demonstrating specificity of radiolabeled antibody immunoreactivity (Figure 1).

### ^64^Cu-NOTA-Bevacizumab Biodistribution and Imaging Indicates Enhanced Renal Tumor Localization

**Figure 2:**
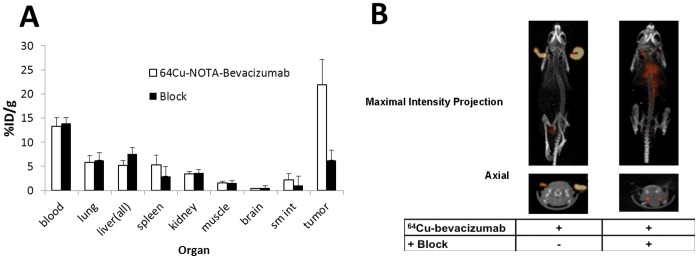
^64^Cu-NOTA-bevacizumab biodistribution. (**A**) Mice with 786-O renal cell carcinoma ear tumors were i.v. injected with 100 µCi of ^64^Cu-NOTA-bevacizumab (n = 4 mice). 24 hours post injection select organs and tumors were collected, weighed, and the amount of activity in each tissue was assessed in a gamma-counter. The decay-corrected % injected dose (ID)/g tissue was calculated for each organ. A blocking dose of 100 µg unlabeled bevacizumab markedly and specifically reduced tumor tracer uptake with no detectable effect on host organ tracer accumulation. (**B**) Mice with 786-O renal cell carcinoma ear tumors were administered 100 µCi of ^64^Cu-NOTA-bevacizumab i.v. in the absence (left panel), or presence (right panel), of 100 µg unlabeled antibody. Twenty min static scans were acquired 24 hr post-injection.

To test ^64^Cu-NOTA-bevacizumab avidity in a tumor with marked VEGF expression, biodistribution studies were performed in athymic nude mice bearing 786-O heterotopic ear tumors Figure 2A and B). The circulating level of ^64^Cu-bevacizumab in the blood was 9.3±3.8% injected dose per gram (ID/g). Tumor uptake was 22.0±5.3% ID/g whereas muscle uptake was only 1.1±0.4% ID/g. Spleen, lung, liver, and kidney uptake were also low at 5.8±2.5%, 4.8±1.7%, 5.6±1.4%, and 2.6±0.6%, respectively. Thus even though the mice bore two tumors, one on each ear, uptake in the single ear tumor sampled was significantly robust compared to host organs. Addition of a 100 µg blocking dose of unlabeled bevacizumab significantly inhibited tumor ^64^Cu-NOTA-bevacizumab uptake demonstrating radiotracer specificity in vivo (Figure 2A). Small animal PET imaging demonstrated high tumor uptake of ^64^Cu-NOTA-bevacizumab (SUV 10.6) (Figure 2B). As a further test of specificity, we administered a 10-fold excess iv blocking dose of bevacizumab in conjunction with the ^64^Cu-NOTA-bevacizumab PET tracer. The unlabeled blocking dose diminished tumor tissue uptake three-fold (Figure 2B). Remarkably, the blocking dose abrogated tumor image detection (6.3±2.1%), with residual signal emanating from the blood tracer pool (Figure 2B).

### RAD001 Diminishes Renal Tumor Target Phosphorylation and VEGF Content

**Figure 3:**
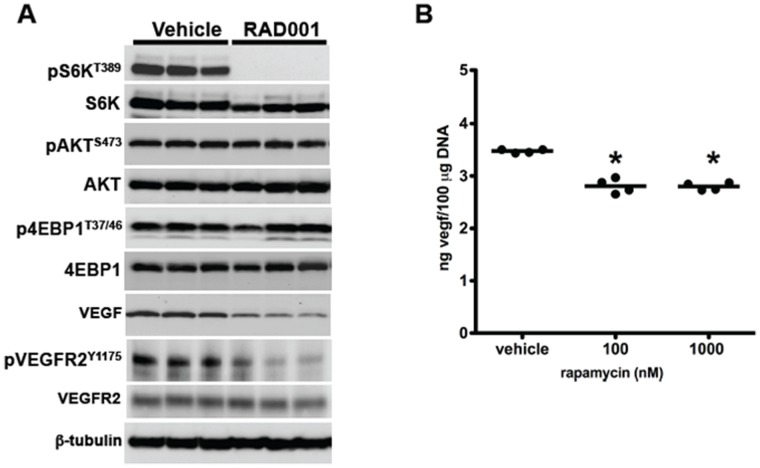
mTORC1 signaling and VEGF secretion in response to RAD001. (**A**) RAD001 abrogated mTORC1-mediated ribosomal protein S6 kinase phosphorylation in tumor lysates without affecting mTORC1-mediated 4E-BP1 phosphorylation. Tumoral VEGF content was decreased by 60% and accompanied by a commensurate similar decrease in VEGFR2 phosphorylation. The immunoblot data were normalized to the unphosphorylated total protein for each target and to β-tubulin. Each lane is lysate from one tumor on one mouse. (**B**) ELISA analysis of conditioned media from RAD001 treated 786-O cultured cells, n = four independent cultures per treatment group, demonstrated a 20% drop in secreted VEGF protein level. The ELISA data were normalized to total cellular DNA to correct for RAD001-mediated decreases in total cell protein. *p<0.0001.

Prior to testing ^64^Cu-NOTA-bevacizumab PET therapeutic response imaging, a cohort of mice was treated daily with RAD001, 10 mg/kg, or vehicle, to determine the rapalog’s effect on tumor mTOR signaling and VEGF content. Fourteen days of RAD001 produced undetectable phosphorylation of the mTOR specific threonine 389 site of ribosomal protein S6 kinase (Figure 3). In contrast to previous work with human cell lines demonstrating rapalog negative feedback PI3K-mTORC2 induction [33], there was a 15% reduction AKT phosphorylation at serine 473 (pAKT^S473^), an mTORC2 target, consistent with rapalog-mTOR sequestration [34]. RAD001 treatment decreased tumoral VEGF protein expression by 60% with a concomitant 60% reduction in VEGFR2 phosphorylation, the principle endothelial cell VEGF signaling receptor as determined by normalization to the respective tubulin loading controls (Figure 3A). To test the cell autonomous effects of rapalogs on 786-O VEGF secretion, conditioned media from rapamycin and vehicle-treated cultures were analyzed for VEGF content. Rapamycin caused a 20% drop in secreted VEGF compared to control (Figure 3B). This partial response of 786-O cells could be due to inability of rapamycin to inhibit key mTORC1 targets regulating translation of “weak mRNAs”, such as 4E-BP1 [13] (Figure 3). There are several explanations for the more pronounced decrease in VEGF levels in the immunoblots versus ELISA analysis. As ELISA measured secreted VEGF it is possible that VEGF was secreted and prior to initiation of rapamycin treatment, therefore the drug only inhibited the translation and secretion of newly synthesized growth factor in cultured cells. In addition, rapamycin may effect the cellular VEGF secretion pathway in a distinct mechanism compared with VEGF mRNA translational regulation. More likely, was the pronounced difference between cell culture wherein nutrient provision is uniform, despite serum starvation, versus the tumor microenvironment in intact mice potentially possessing perfusion mismatches or arteriovenous shunting.

### ^64^Cu-NOTA-Bevacizumab Biodistibution Alterations in Response to RAD001

**Figure 4:**
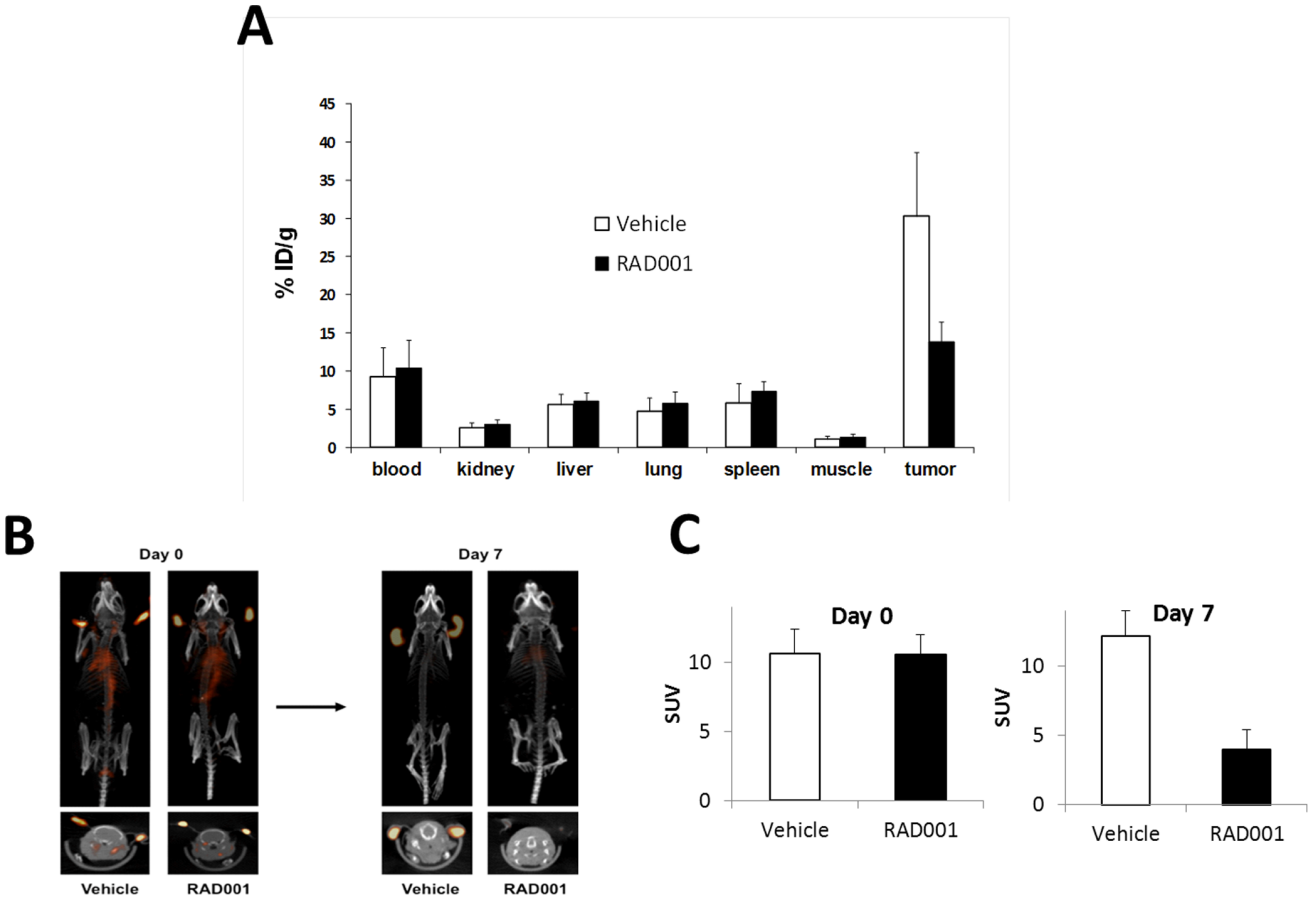
Pre and post-treatment microPET/CT Study. (**A**) Biodistribution of ^64^Cu-NOTA-bevacizumab in mice (n = 4 mice per group) bearing 786-O renal cell carcinoma tumors after treatment with RAD001 or vehicle. (**B**) Mice bearing 786-O renal cell carcinoma ear tumors were scanned at baseline following i.v. injection of 100 µCi ^64^Cu-NOTA-bevacizumab (n = 4). RAD001 was then administered for 7 days and the scans repeated. There was a marked decreased in ^64^Cu-NOTA-bevacizumab signal in the RAD001 compared to the vehicle control treated mouse. (**C**) Diminution of standard uptake value (SUV) in the RAD001 compared to vehicle treated mice.

**Figure 5:**
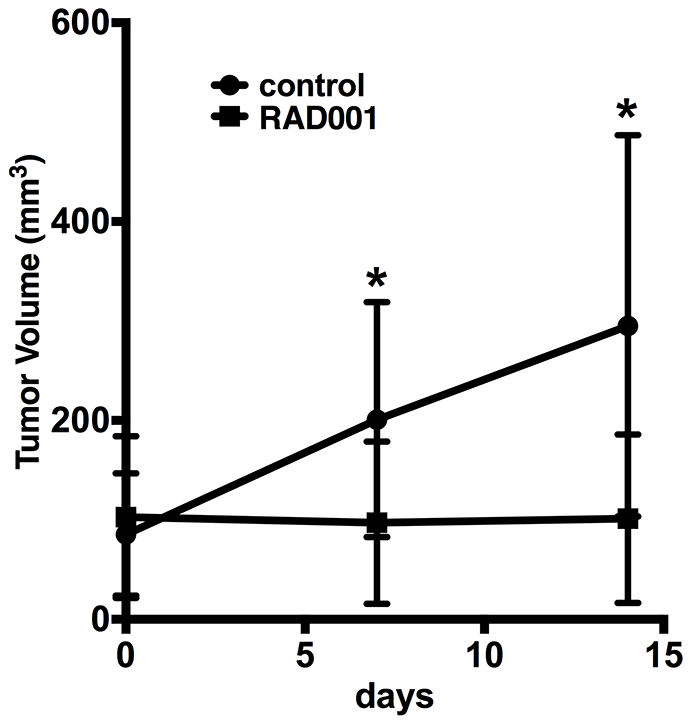
RAD001-mediated 786-O growth inhibition. Tridimensional ear tumor growth determination reveals that RAD001 (▪) essentially prevents the growth of ear tumors (n = 9 mice bearing 18 bilateral tumors) compared to linear growth in vehicle controls (•) (n = 7 mice bearing 14 bilateral tumors).

Next, we investigated whether ^64^Cu-NOTA-bevacizumab PET imaging could detect RAD001-mediated tumoral VEGF content decreases in a mouse model of RCC. Biodistribution studies were performed at 7 rather than 14 days of RAD001 administration to test whether VEGF uptake was dissociated from significant alterations of tumor growth (Figure 4A). RAD001 significantly decreased tumor ^64^Cu-NOTA-bevacizumab uptake two-fold, vehicle treated tumors, 30.3±8.3%, RAD001 13.8±2.6% ID/g respectively, *p*<0.001. MicroPET/CT imaging studies performed in a parallel cohort of tumor bearing mice (Figure 4B and C) revealed a three-fold diminution of tumoral ^64^Cu-NOTA-bevacizumab signal intensity, SUV 4.0, compared vehicle-treated mice, SUV 12.2. Tumor growth response analysis in another parallel cohort of RAD001 and vehicle treated mice (see Materials and Methods) revealed that RAD001 prevented tumor size expansion during the 14 days of treatment as determined by linear regression analysis wherein the slope of the vehicle group growth increase was 14.98 (p = 0.0001), whereas the slope of the RAD001 treated group was 0.20 (p = 0.92). Two-way ANOVA analysis also revealed a significant difference in tumor sizes according to measurement day and vehicle versus drug treatment. Unfortunately, differential, RAD001 tumor volume diminution had already reached statistical significance by day 7, as determined by one-way ANOVA, p = 0.003 for the day 0-day 7 interval or single time point analysis of day 7 tumor size between the vehicle and RAD001 groups, Mann-Whitney U-test, p = 0.013, preventing determination of the predictive value of VEGF-PET imaging at this time point (Figure 5).

## Discussion

In this study, we successfully synthesized and evaluated ^64^Cu-NOTA-bevacizumab for imaging of VEGF levels in RCC tumor xenografts. This compound was stable, specifically accumulated in tumors, and could be blocked by the addition of unlabeled bevacizumab. Additionally, a RAD001-mediated decrease in VEGF expression was clearly visualized by PET imaging using ^64^Cu-NOTA-bevacizumab. Tracer uptake diminution was concomitant with static effect of RAD001 on tumor growth. Thus, ^64^Cu-NOTA-bevacizumab may be a novel surrogate biomarker for disease stabilization mediated by rapalog or mTOR kinase inhibitor therapies.

Traditionally solid tumor therapeutic response is scored based on tumor dimensional reduction, either measured physically, radiographically, or by CT and MRI. The Response Evaluation Criteria in Solid Tumors (RECIST) and its more recent RECIST1.1 version provides a standardized methodology for efficacy comparisons across drugs and studies. However, the emergence of molecularly targeted therapies challenges RECIST as an efficacy benchmark [35]. When molecularly targeted therapies are effective, they principally produce stable disease (SD) or partial response (PR) with minimal changes in tumor size. Limitations of RECIST for molecular therapy efficacy determination motivates development of CT, MRI, and ultrasound imaging algorithms and techniques tailored in particular for tumor vascular targeting drugs. These algorithms are designed to detect changes in overall tumor vascular density, angiogenic factor mediated vascular leakage, and tumor vascular flow [36].

Another approach to assess therapy efficacy is PET. Tumors are characterized by enhanced glucose and thymidine uptake in response to genetic dysregulation of metabolism and proliferation [37]. The radiotracers ^18^F-fluorodeoxyglucose (FDG) and ^18^F-fluorothymidine (FLT) [38]–[40], image tumor glucose uptake and DNA synthesis detecting primary tumor and metastasis and monitoring therapeutic response. Both FDG and FLT PET can reveal therapeutic efficacy prior to tumor volume changes. These two PET tracers are challenged by the fact that some cancers such as RCC (and prostate) are predominantly FDG insensitive [41], and FLT underestimates proliferation in tumors with activated pyrimidine de-novo synthesis or salvage pathway upregulation [42]. However, in the minority of FDG positive RCCs, PET detects tumor response earlier than size reduction and predicts better progression free survival (PFS) [14].

Despite limitations in cancers such as RCC, a new dimension in PET has become apparent in the last few years. “ImmunoPET” tracers are antibody-radionuclide conjugates binding to proteins either attached to the cell surface, or secreted into the tumor microenvironment [43]. Using bifunctional chelators such as the NCS (isothiocyanate) or NHS (N-Hydroxysuccinimide) forms of 1,4,7,10-tetraazacyclododecane-1,4,7,10-tetraacetic acid (DOTA) or 1,4,7-triazacyclononane-N,N’N”-triacetic acid (NOTA), radionuclide metal ions can be complexed in the chelator cores while covalent bonds are formed with free lysine groups on antibodies or peptides [44]. The power of this approach is that DOTA/NOTA chelated PET tracers can be conjugated to antibodies specific for a repertoire of angiogenic factors. As VEGF is the principal angiogenic factor produced by RCC (and most other cancers as well), this angiogenic factor has been explored as an imaging target. Prior studies have evaluated the use of radiolabeled bevacizumab for PET imaging of VEGF expression. For example, bevacizumab has been radiolabeled with ^89^Zr [26] and ^86^Y [27] for preclinical imaging of ovarian xenograft models. Despite the ability of these agents to image VEGF, the high energy and abundance of the gamma decay from these radionuclides are an exposure concern compared to ^64^Cu which has a much lower high energy gamma decay abundance. Paudyal *et al.* evaluated ^64^Cu-DOTA- bevacizumab for preclinical VEGF expression imaging in colorectal xenografts [28]. Liver uptake was relatively high,17.2% ID/g, in contrast to our ^64^Cu-NOTA-bevacizumab tracer where it was 4.8% ID/g. An explanation for the differential liver uptake could be the higher ^64^Cu binding affinity of NOTA compared to DOTA. Nagengast *et al*. demonstrated the ability of ^89^Zr-radiolabeled ranibizumab to measure dynamic changes in VEGF expression following sunitinib treatment, a multi-tyrosine kinase inhibitor also used in metastatic renal cancer [26]. However, low tumor (∼5–6% ID/g) and high kidney uptake [26] may limit the use of radiolabeled ranibizumab in intra-abdominal tumors. In contrast, we found relatively high ^64^Cu-NOTA-bevacizumab accumulation (∼30% ID/g) in untreated renal cancer xenografts tumors in comparison to normal organs such as liver, kidney, GI tract, and bladder, similar to other groups using this tracer [29]. Our increased VEGF signal-to-noise facilitated tumor imaging enhancement and its rapalog-mediated decrease.

A potential limitation of our study is that bevacizumab binds only to human and not to mouse VEGF. As such, host-derived VEGF sources such as stromal fibroblasts, myeloid cells, and vascular supporting cells were undetectable [45]–[47]. Therefore, in humans, the potential exists for increased tumor to background ratio of ^64^Cu-NOTA-bevacizumab uptake due to VEGF production from multiple sites in the tumor microenvironment. Moreover, inhibition of VEGF secretion from tumor associated myeloid cells could be of near equal therapeutic importance as malignant cellular VEGF production. In addition, the ability of humanized bevacizumab to detect VEGF in the heart, lung, kidney, and brain could be important predictors of VEGF signaling inhibitor toxicities such as proteinuria, pulmonary hemorrhage, and cardiotoxicity [48], [49]. Signal to noise and utility of VEGF content imaging in normal organs, for therapy toxicity monitoring will be interesting venues for investigation in patient studies.

Although rapid tumor growth prevented assessment of the predictive ability, of ^64^Cu-NOTA-bevacizumab PET, the concordance of imaging and tracer biodistribution with our preclinical disease stabilization suggests a translational role for immunoPET during patient selection for molecularly targeted therapies. mTORC1 regulation of “weak” mRNA translation encompasses secreted growth factors many of which are angiogenic factors [50]. Therefore, VEGF, or an expanded repertoire of immunoPET tracers, cognate for other secreted molecules that are mTOCR1 targets, could detect patients that may be inhibitor sensitive. This potential ability for rapid patient selection based on functional PET is particularly important as substantial percentages of solid tumors either possess signaling pathways bypassing the mTORC1 translational requirement, or upregulate or amplify genes encoding downstream mTORC1 translational effectors [50]. In addition, breakthrough from mTORC1-mediated stable disease to progressive disease could also be detected prior to measurable tumor expansion. Conversely, ^64^Cu-NOTA-bevacizumab PET could also be used to track elevations of tumor VEGF during VEGFR inhibitor therapies. Drug dosage could then be increased in those patients to match the increase in tumor production. In addition, libraries of immunoPET tracers could potentially pinpoint which compensatory angiogenic factor(s) are induced during mTORC1 or VEGFR inhibitor therapies and an appropriate alternative pathway-specific inhibitor deployed. As such, the concept of immunoPET reporting on tumor angiogenic factor, chemokine, or cytokine production offers great potential for therapy tailored personalized medicine. However, in order to supplant or even augment criteria such as RECIST that was established from data acquired for large patient populations, PET studies will similarly need to be tested in large patient cohorts preferably encompassing several different tumor histotypes.
